# Effect of *Cordyceps militaris* Powder Prophylactic Supplementation on Intestinal Mucosal Barrier Impairment and Microbiota-Metabolites Axis in DSS-Injured Mice

**DOI:** 10.3390/nu15204378

**Published:** 2023-10-16

**Authors:** Shujian Wu, Zaoxuan Wu, Ye Chen

**Affiliations:** 1 Shenzhen Clinical Research Center for Digestive Disease, Integrative Microecology Clinical Center, Shenzhen Key Laboratory of Gastrointestinal Microbiota and Disease, Shenzhen Technology Research Center of Gut Microbiota Transplantation, Shenzhen Hospital, Southern Medical University, Shenzhen 518100, China; sjwu@stu2018.jnu.edu.cn; 2 State Key Laboratory of Organ Failure Research, Department of Gastroenterology, Nanfang Hospital, Southern Medical University, Guangzhou 510080, China; wzx1914458273@163.com

**Keywords:** *Cordyceps militaris*, ulcerative colitis, gut microbiota, metabolites, intestinal mucosal barrier

## Abstract

Ulcerative colitis (UC) is a chronic and recurrent inflammatory disease with an unknown pathogenesis and increasing incidence. The objective of this study is to investigate the impact of prophylactic treatment with *Cordyceps militaris* on UC. The findings demonstrate that prophylactic supplementation of *C. militaris* powder effectively mitigates disease symptoms in DSS-injured mice, while also reducing the secretion of pro-inflammatory cytokines. Furthermore, *C. militaris* powder enhances the integrity of the intestinal mucosal barrier by up-regulating MUC2 protein expression and improving tight junction proteins (ZO-1, occludin, and claudin 1) in DSS-injured mice. Multiomics integration analyses revealed that *C. militaris* powder not only reshaped gut microbiota composition, with an increase in *Lactobacillus*, *Odoribacter*, and *Mucispirillum*, but also exerted regulatory effects on various metabolic pathways including amino acid, glyoxylates, dicarboxylates, glycerophospholipids, and arachidonic acid. Subsequent analysis further elucidated the intricate interplay of gut microbiota, the intestinal mucosal barrier, and metabolites, suggesting that the microbiota–metabolite axis may involve the effect of *C. militaris* on intestinal mucosal barrier repair in UC. Moreover, in vitro experiments demonstrated that peptides and polysaccharides, derived from *C. militaris*, exerted an ability to change the gut microbiota structure of UC patients’ feces, particularly by promoting the growth of *Lactobacillus*. These findings suggest that regulatory properties of *C. militaris* on gut microbiota may underlie the potential mechanism responsible for the protective effect of *C. militaris* in UC. Consequently, our study will provide support for the utilization of *C. militaris* as a whole food-based ingredient against the occurrence and development of UC.

## 1. Introduction

With rapid diets and lifestyle changes, Inflammatory Bowel Disease (IBD) has progressively become a growing challenge to public health in the world. Ulcerative colitis (UC), a subtype of IBD, is characterized by clinical symptoms such as abdominal pain and bloody diarrhea, significantly influencing the quality of life. At the turn of the 21st century, the prevalence rates of UC were recorded at 505 per 100,000 in Norway (Europe), 286 per 100,000 in the USA (North America), 57 per 100,000 in Japan (Eastern Asia), and 196 per 100,000 in Barwon (Oceania) [1]. The global incidence and prevalence of UC are rising, while its pathogenesis remains unclear [1,2]. Despite the availability of several biological agents for the treatment of UC, response and remission rates remain unsatisfactory in approximately 60% of cases [3]. Therefore, it is necessary to explore more effective ways and approaches to alleviate UC.

Increasing evidence has revealed a strong association between gut microbiota and the intestinal mucosal barrier in relation to UC [4,5,6]. The intestinal mucosal barrier plays a critical role in preventing the entry of and responding to harmful contents [7]. Numerous studies have reported that intestinal mucosal barrier dysfunction is an early event in UC [8]. Moreover, existing data clearly imply that gut microbiota dysbiosis plays a central role in the pathogenesis of UC [6,9,10]. The integrity and function of the intestinal mucosal barrier may be damaged by gut microbiota dysbiosis, thereby promoting the proliferation of the pathogenic bacteria and secretion of enterotoxins, resulting in an increase in the intestinal permeability and intestinal immune dysregulation, eventually leading to the onset of chronic intestinal inflammation [6]. Therefore, it is a potential therapeutic target for UC to balance gut microbiota and regulate the intestinal mucosal barrier function.

Most mushrooms, rich in biocomponents, are not easily digestible and/or absorbed in the upper gastrointestinal tract, but are excellent sources of prebiotics to interact with gut microbiota [11]. *Cordyceps militaris* (*CM*), a medicinal mushrooms, is cultured *Cordyceps sinensis* mycelium, which is widely used in many Asian countries [12]. Previous studies have demonstrated that *CM* powder exhibited an ability to regulate the immune system in humans [13] and protect mice from triptolide-induced acute hepatotoxicity [12]. Recent studies also highlighted the potential of active constituents from *CM* to ameliorate type 2 diabetes mellitus, obesity, and neurodegenerative diseases, through modulating gut microbiota and improving intestinal mucosal structure damage [14,15,16]. However, multiple biocomponents of *CM* may have potential synergistic effects, and it remains unclear whether *CM* can alleviate UC by regulating the intestinal mucosal barrier and gut microbiota. Therefore, this study aims to investigate the prophylactic supplementation effect of *CM* on DSS-induced UC in mice and its impact on gut microbiota structure, through in vitro fermentation of UC patients’ feces. We attempt to explore the effects of *CM* on the explicit mechanisms of gut microbiota, the intestinal mucosal barrier, and UC.

## 2. Materials and Methods

### 2.1. Animals and Treatments

**Figure 1:**
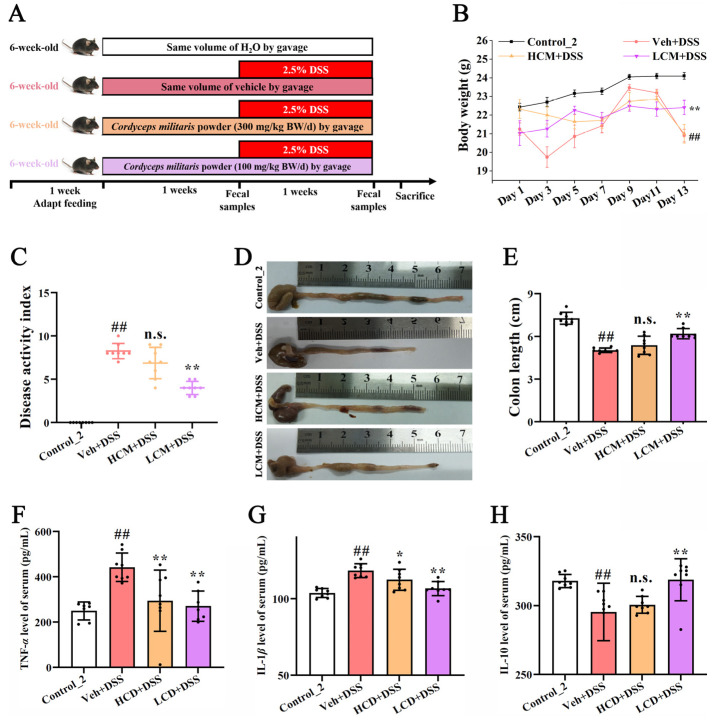
Effects of *CM* powder on the symptoms of mice with DSS-induced ulcerative colitis (*n* = 8). (**A**) Schematic representation of experimental protocol. (**B**) Body weight. (**C**) DAI score. (**D**,**E**) Colon length. (**F**–**H**) The levels of TNF-*α*, IL-1*β*, and IL-10 in the serum. ##, *p* < 0.01 versus control group; *, *p* < 0.05 versus model group; **, *p* < 0.01 versus model group; n.s., no significant difference (*p* > 0.05).

Thirty-two male C57BL/6 mice, aged 6–7 weeks and weighing 20 ± 2 g, were obtained from Sibei Fu Biotechnology Co., Ltd. (Beijing, China), and housed in an environment with a temperature of 24 ± 2 °C and a light/dark cycle of 12 h, with food and water provided ad libitum. After 1 week for adaptation, the mice were randomly divided into four groups (*n* = 8). *CM* and dextran sodium sulfate (DSS) were obtained from Guangdong Fudonghai Pharmaceutical Co., Ltd. (Zhanjiang, China) and MP Biomedicals Co., Ltd. (Irvine, CA, USA), respectively. As illustrated in Figure 1A, the control group was treated with sterile water, whereas the high-dose *CM* group (HCM + DSS) and low-dose *CM* group (LCM + DSS) received *CM* powder at 300 mg/kg BW/day and 100 mg/kg BW/day (using 0.1% hydroxymethyl cellulose as a vehicle) by oral gavage once daily for 14 days, respectively. Meanwhile, the model group (Veh + DSS) received the same amount of 0.1% hydroxymethyl cellulose once daily. From days 7 to 14, mice in all groups except the control group were administered 2.5% (*w*/*v*) DSS in drinking water to induce colitis. At the end of the experiment, mice were anesthetized with ether, and blood samples were collected by enucleation of the eyeballs. The samples were then centrifuged at 4 °C and 3000× *g* for 20 min to obtain the serum. Subsequently, the mice were then euthanized by cervical dislocation. The colon length was measured, and distal colon tissues (approximately 1.5 cm) were fixed with 4% paraformaldehyde before the tissue sections were prepared.

### 2.2. Histopathological Examination and Evaluation of Colons

The distal colon was dehydrated and embedded in paraffin, then cut into 5-μm slices and stained with a hematoxylin and eosin (H&E) stain. Histopathological changes were examined using a microscope (NIKON, Tokyo, Japan).

### 2.3. Disease Activity Index and Histological Score

The disease activity index (DAI) score and histological score (HS) of the colons were calculated according to Table 1 and Table 2.

### 2.4. Cytokine Analysis of Serum

The levels of IL-1*β*, TNF-*α*, IL-6, and proteins were analyzed using the commercially available kits, according to the manufacturers’ instructions (Mlbio, Shanghai, China).

### 2.5. Immunofluorescence Assessment

The paraffin sections of the colon tissues were obtained in the same way as the H&E stain. After dewaxing and hydration, 3 μm thick slices were subjected to antigen repair and 5% BSA closure. The sections were then incubated overnight at 4 °C with an anti-rabbit MUC2 antibody (Abclonal, Woburn, MA, USA, A14659), anti-rabbit occludin (CST, 91131S), anti-rabbit claudin 1 antibody (Proteintech, Rosemont, IL, USA, 13050-1-AP), and an anti-rabbit ZO-1 antibody (Proteintech, 21773-1-AP), respectively. Subsequently, they were incubated for 1 h at 37 °C with Alexa Fluor 488-labelled goat antirabbit secondary antibody (Beyotime, A0423), and covered with an anti-fluorescence quenching sealing solution containing DAPI (Beyotime, Shanghai, China) for 1 h at room temperature. Finally, the images were captured using a fluorescence microscope and analyzed using ImageJ software (1.6).

### 2.6. 16S rRNA Gene Sequencing for Fecal Matter

Fecal samples were collected from each mouse at 7 days (time 1, T1), which were labelled as control_1 group, Veh group, HCM group, and LCM group. Fecal samples were also collected from each mouse at 14 days (time 2, T2), which were labelled as control_2 group, Veh + DSS group, HCM + DSS group, and LCM + DSS group.

The genomic DNA of the mouse fecal samples at T1 and T2 was obtained using the E.Z.N.A. soil DNA kit (Omega Bio-Tek, Norcross, GA, USA). The extracted DNA was amplified by PCR in the V3-V4 hypervariable region. The primers were 341F (5′-CCTAYGGGRBGCASCAG-3′) and 806R (5′-CCTAYGGGRBGCASCAG-3′). Sequencing was performed using the Illumina MiSeq PE300 platform/NovaSeq PE250 platform (Illumina, San Diego, CA, USA), according to the standard procedure of Majorbio Bio-Pharm Technology Co., Ltd. (Shanghai, China).

### 2.7. Untargeted Fecal Metabolomics Analysis

Untargeted metabolomic profiling of feces at T2 was conducted by Biotree Biotech Co., Ltd. (Shanghai, China). A 25 mg sample was mixed with a 500 μL extraction solvent (acetonitrile/methanol, 1:1) containing an isotopically labelled internal standard. Next, the mixture was vortexed for 30 s and subjected to ultrasound treatment for 10 min. After resting at −40 °C for 1 h, the supernatant was obtained by centrifugation at 13,800× *g* for 15 min at 4 °C. The supernatant was then analyzed by LC-MS/MS with a UHPLC system (Vanquish, Thermo Fisher Scientific, Waltham, MA, USA), coupled to an Orbitrap Exploris 120 mass spectrometer (Orbitrap MS, Thermo Fisher Scientific, Waltham, MA, USA), utilizing a Waters ACQUITY UPLC BEH Amide column (2.1 mm × 100 mm, 1.7 μm). The mobile phases of A and B were water and acetonitrile, respectively. The temperature and injection volume were set at 4 °C and 2 μL, respectively. The quality control (QC) sample was prepared by mixing an equal aliquot of the supernatant of samples. The Betaine-(trimethyl-d9) hydrochloride, L-leucine-5,5,5-d3, Trimethylamine-d9 N-Oxide, Hippuric acid-d5, [13C3]-L-(+)-sodium lactate, and L- leucine -5,5,5-d3 was the internal standard.

The raw data were obtained under both positive and negative ion models, and then converted to the mzXML format using ProteoWizard, processed with an in-house program developed using R and based on XCMS for peak detection, extraction, alignment, and integration. The metabolites were authenticated by searching an in-house MS2 database (Biotree DB (v2.1)). The metabolites were annotated when the MS2 score was >0.3.

### 2.8. In Vitro Fermentation of Peptides and Polysaccharides from CM

The peptides and polysaccharides were obtained from *CM* [14,15,17,18]. The *CM* powder was extracted with water. Next, the supernatant was precipitated using ethanol and ammonium sulfate. Then, it was dialyzed and lyophilized to obtain the proteins and polysaccharides. The proteins and polysaccharides were further digested in vitro using simulated gastrointestinal digestion with pepsin and pancreatin.

Four volunteers of patients with active UC (2 females and 2 males, aged 30–40 years, China) provided fecal samples. The fresh feces (5.0 g with anaerobic bags) were mixed with PBS (pH = 7, 0.15% cysteine) at a ratio of 1:4 (g/mL), and then centrifuged at 500× *g* for 5 min at 4 °C. Afterwards, the supernatant, as a bacterial suspension for fermentation, was collected and stored at −80 °C before use.

The fermentation process and the preparation of the in vitro fermentation medium were performed according to a previous method. Briefly, 1 mL bacterial suspension was mixed with 4 mL culture medium in each anaerobic tube. The peptides and polysaccharides were added into the anaerobic tubes at a concentration of 5 mg/mL, respectively. The fermentation process was performed under anaerobic conditions with 37 °C and 60 rpm. Samples were collected at 0, 6, 12, and 24 h by centrifugation (12,000× *g*, 12 min). The precipitation was analyzed for 16S rRNA gene sequencing.

### 2.9. Statistical Analysis

Data were presented as means ± SD or SEM. Statistical analysis was conducted with IBM SPSS 19.0 software, using a one-way analysis of variance (ANOVA) test with Duncan’s multiple range test (*p* < 0.05). Graph design was performed using GraphPad Prism 8.0.2 software and Origin 9. The intensity of the fluorescence was analyzed by using the Image J software.

## 3. Results

### 3.1. CM Powder Alleviated DSS-Induced UC in Mice

After two weeks (Figure 1), compared to the control_2 group, the body weights of the DSS-injured mice were reduced by 13.28%, while the low dose *CM* powder treatment (7.02%) significantly inhibited weight loss (*p* < 0.01) (Figure 1B). As the DAI score (Figure 1C) and colon length (Figure 1D,E) showed, a significant increase in DAI score and an obvious reduction in colon length were observed in the Veh + DSS group (*p* < 0.01). Notably, the adverse symptoms in the LCM + DSS group were significantly improved (*p* < 0.01). Moreover, pro-inflammatory cytokines (TNF-*α* and IL-1*β*) and anti-inflammatory cytokines (IL-10) played an important role in UC [19]. A reduction in TNF-*α* and IL-1*β* and an increase in IL-10 were observed in the LCM + DSS group (Figure 1F–H). Collectively, these findings implied that *CM* powder could alleviate the disease symptoms in DSS-injured mice.

### 3.2. CM Powder Enhanced Intestinal Mucosal Barrier Function

**Figure 2:**
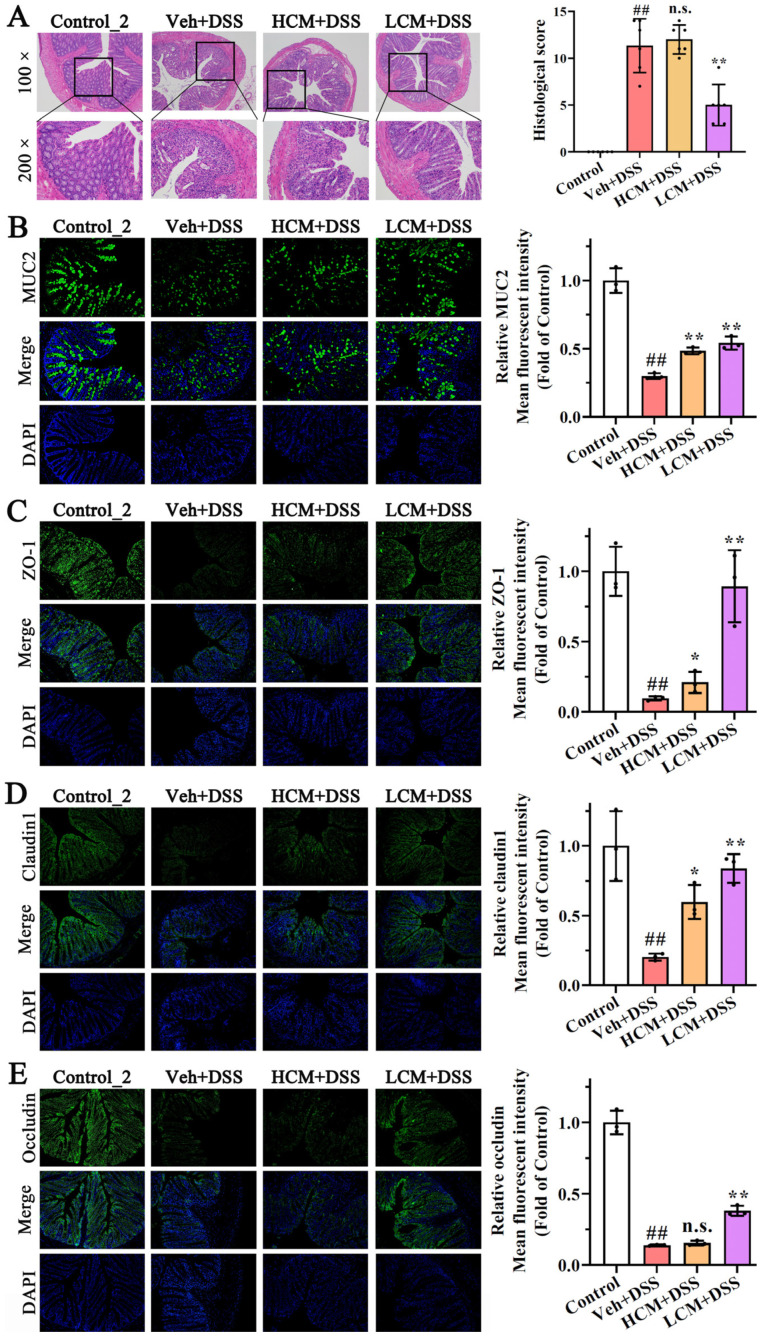
Effects of *CM* powder on intestinal mucosal barrier in DSS-induced ulcerative colitis mice. (**A**) Representative images of H&E staining (magnification of 100× and 200×) and its histopathological evaluation (*n* = 8). (**B**–**E**) Representative images (magnification of 200×) of immunofluorescence staining of MUC2, ZO-1, claudin 1, and occludin, and its mean fluorescent intensity (*n* = 3). All images were taken at the same scale. ##, *p* < 0.01 versus control group; *, *p* < 0.05 versus model group; **, *p* < 0.01 versus model group; n.s., no significant difference (*p* > 0.05).

The intact intestinal mucosal barrier plays an important role in preventing bacterial invasion and physical damage, which is critical in UC [20]. As shown in Figure 2A, there were no histopathological changes in the control_2 group with an intact mucosa and abundant goblet cells. In contrast, severe damage to the epithelial surface, mucosal inflammation, extensive inflammatory cell infiltration, goblet cell exhaustion, and crypt distortion were observed in the DSS-treated mice. However, treatment with the low dose of *CM* powder attenuated these pathological changes, indicating that the colonic structure damages were repaired in DSS-induced UC mice.

The immunofluorescence analysis showed that the levels of MUC2, ZO-1, claudin 1, and occludin were markedly down-regulated in DSS-induced UC mice (*p* < 0.01), whereas *CM* powder treatment increased the levels of MUC2, ZO-1, claudin 1, and occludin in the distal colon both of the HCM + DSS group and the LCM + DSS group (Figure 2). Notably, there were no statistically significant differences in the HS and the level of occludin protein between the Veh + DSS group and the HCM + DSS group. Compared to the HCM + DSS group, a higher level of MUC2, ZO-1, and claudin 1 was observed in the LCM + DSS group. These results suggest that the *CM* powder treatment had an excellent protective effect on the intestinal mucosal barrier function of DSS-injured mice, especially the low dose *CM* powder treatment.

### 3.3. CM Powder Intervention Changed the Fecal Microbiota Composition

**Figure 3:**
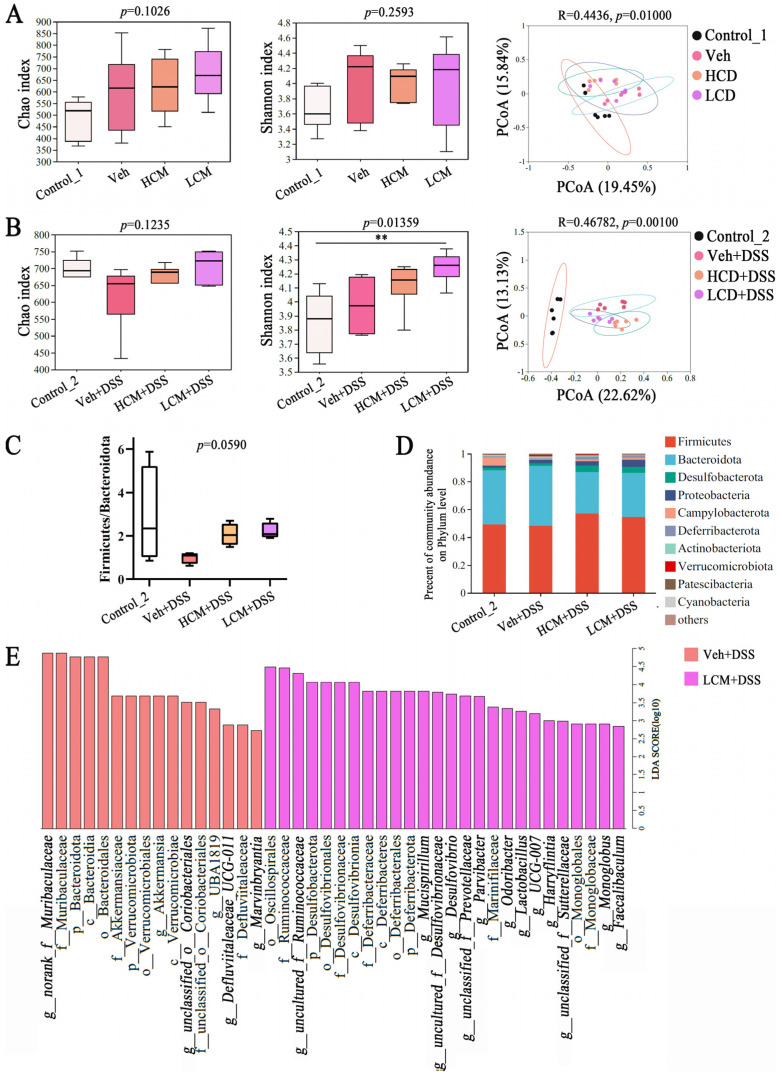
Effects of *CM* powder on gut microbiota at T1 and T2 (*n* = 6). (**A**) Chao index, Shannon index, and the principal coordinate analysis (PCoA) at the OTU level of different groups at T1. (**B**) Chao index, Shannon index, and PCoA at the OTU level of different groups at T2. (**C**) The ratio of Firmicutes/Bacteroidetes of different groups at genus level of group at T2. (**D**) The community bar plots at the phylum level at T2. (**E**) The LEfSe analyses from genus to phylum at the genus level at T2. **, means significant difference between two group (*p* < 0.01).

A total of 3,622,264 and 3,538,725 raw reads were obtained from 24 samples at T1 and T2, respectively. After identification and removal of the chimeric sequences, 1581 and 1834 OTUs with a 97% similarity level were obtained, respectively. The rank abundance (Figure S1A), Shannon index, and rarefaction curves (Figure S1B), and the coverage index (Figure S1C,D) indicated that the sequencing depth covers most of the bacterial diversity, including rare new phylotypes. Compared to the control_1 group, the richness (Chao index, Figure 3A) and diversity (Shannon index, Figure 3A) were enhanced by hydroxymethyl cellulose and *CM* powder treatment at T1. However, the different groups were not separate, but rather intermingled with the overall region in principal coordinate analysis (PCoA) (Figure 3A), indicating that there was no statistical difference between the groups. The results suggested that *CM* powder could affect the gut microbiota structure but tended to be similar to control_1 group at T1. After the mic were exposed to DSS, compared to the control_2 group, the richness was decreased in the Veh + DSS group; the control_2 group and the Veh + DSS group separated from each other in PCoA, outside the overall region at T2 (Figure 3B), indicating a significant change in the gut microbiota composition in DSS-induced UC. On the other hand, *CM* powder can enhance the richness and diversity in DSS-injured mice (Figure 3B), and obvious differences were observed among the Veh + DSS group, HCM + DSS group, and LCM + DSS group (PCoA, Figure 3B). Hence, a prophylactic treatment of *CM* powder can influence the gut microbiota composition in DSS-induced UC.

The OTUs of the HCM + DSS group and the LCM + DSS group were enhanced at T2, compared to DSS-injured mice. There were 261, 107, 162, and 176 unique OTUs in the control_2 group, Veh + DSS group, HCM + DSS group, and LCM + DSS group, respectively (Figure S1E). Additionally, the Firmicutes/Bacteroidetes (F/B) ratio is an important indicator of gut microbiota dysbiosis [15]. The F/B ratio decreased in the Veh + DSS group compared to the control_2 group, while the *CM* powder treatment prevented a decrease in the F/B ratio in the HCM + DSS group and LCM + DSS group (Figure 3C). These findings imply that the *CM* powder treatment effectively improved gut microbiota dysbiosis in UC. At the phylum level and genus level, Firmicutes, Bacteroidota, Desulfobacterota, and Proteobacteria were the most common bacterial phylum in all groups. Among them, Firmicutes and Bacteroidota accounted for more than 90% of the total microbiota in each group at phylum level (Figure 3D). *Norank_f__Muribaculaceae*, *norank_f__norank_o__Clostridia_UCG-014*, *Bacteroides*, *Phascolarctobacterium*, *Lachnospiraceae_NK4A136_group*, and *unclassified_f__Lachnospiraceae* were the topmost abundant microbiota in all experimental groups at the genus level (Figure S1F). The linear discriminant analysis effect size (LEfSe) was performed to uncover the differences between the Veh + DSS group and LCM + DSS group (from the phylum to genus level). The linear discriminant analysis (LDA, score > 2.0) showed that the change in gut microbiota in relative abundance was caused by 16 dominant communities in the Veh + DSS group and 26 dominant communities in the LCM + DSS group (Figure 3E). At genus level, *norank_f__Muribaculaceae*, *Akkermansia*, *Defluviitaleaceae_UCG-011*, and *Marvinbryantia* were abundant in the Veh + DSS group. The low dose *CM* powder intervention significantly altered the abundances of *uncultured_f__Ruminococcaceae*, *Mucispirillum*, *Lactobacillus*, *Desulfovibrio*, *unclassified_f__Prevotellaceae*, *Parvibacter*, *Odoribacter*, *Harryflintia*, *UCG-007*, *unclassified_f__Sutterellaceae*, *Monoglobus*, and *Faecalibaculum* in DSS-injured mice. Therefore, the alteration of gut microbiota using a low dose of *CM* powder may be critical for the alleviation of UC.

### 3.4. CM Powder Modulated the Fecal Metabolome in DSS-Injured Mice

**Figure 4:**
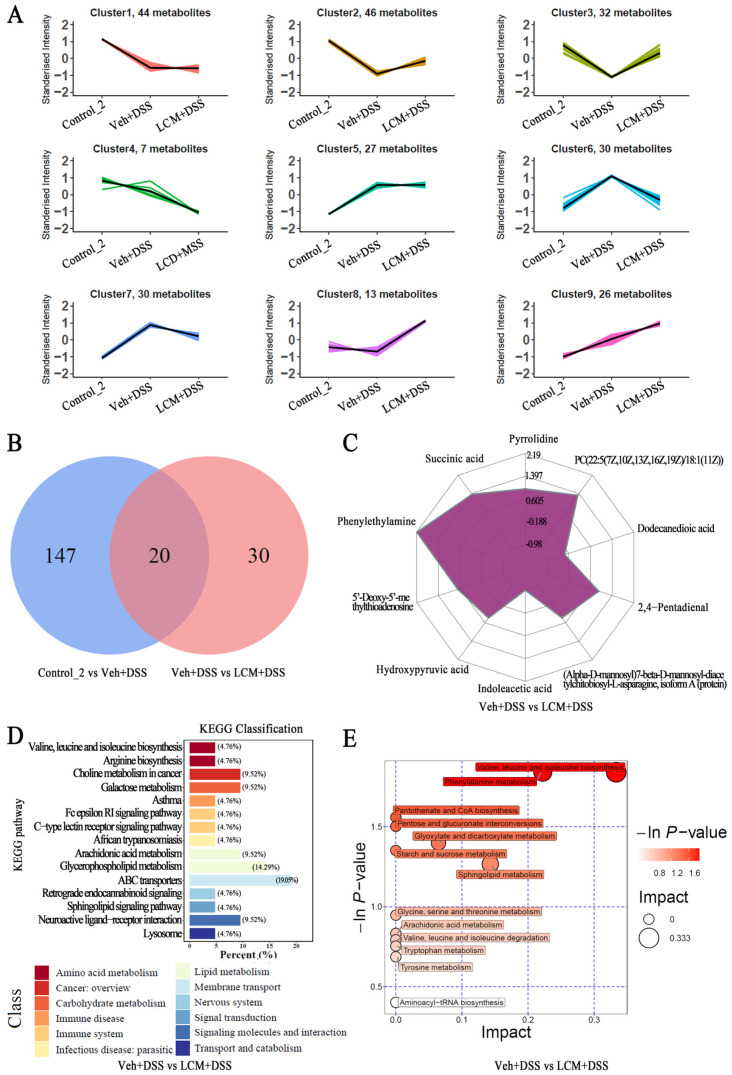
Effects of *CM* powder on metabolites of feces in DSS-induced ulcerative colitis mice at T2 (*n* = 4). (**A**) k-means cluster analysis of differential metabolism. Different colors represent different clusters. (**B**) Venn analysis for differential metabolism. (**C**) Radar chart analysis of differential metabolism for Veh + DSS vs. LCM + DSS. (**D**) KEGG classification of differential metabolism for Veh + DSS vs. LCM + DSS. (**E**) Pathway analysis of differential metabolism for Veh + DSS vs. LCM + DSS.

The fecal metabolome was further analyzed using untargeted metabolomics. The OPLS-DA model showed that there was a good predictability and did not overfit, according to *p* < 0.05 and R2Y values of 0.99 and 0.97 in control_2 vs. Veh + DSS and Veh + DSS vs. LCM + DSS, respectively (Figure S2A). The principal component analysis (PCA) showed an obvious separation trend in control_2 vs. Veh + DSS and Veh + DSS vs. LCM + DSS, implying that significant differences existed in fecal metabolites among the different experimental groups (Figure S2B). Significant differential metabolites were identified based on a threshold of VIP > 1 and *p* < 0.05 from all groups, including lipids and lipid-like molecules (31.37%), organic acids and derivatives (25.64%), organoheterocyclic compounds (13.87%), organic oxygen compounds (7.99%), and others (21.12%) (Figure S2C). The K-means clustering analysis shows that nine distinct clusters of differential metabolites were determined based on the variation tendency, which indicated great differences in metabolites following treatment using DSS and *CM* powder. DSS induced a decreasing tendency in clusters 2, clusters 3, and clusters 8, and an increasing tendency in clusters 6 and clusters 7 compared to control_2, while a low dose *CM* powder treatment reversed the tendency (Figure 4A), revealing that *CM* powder treatment affected the gut microbiota-derived metabolites significantly.

There were 167 and 50 significantly differential metabolites (with accurate names in the database) in control_2 vs. Veh + DSS and Veh + DSS vs. LCM + DSS (Table S1). Compared to the control_2 group, 69 metabolites were up-regulated, while 98 were down-regulated in the Veh + DSS group (Table S1 and Figure S2D). Compared to the Veh + DSS group, 37 metabolites were up-regulated while 13 were down-regulated in the LCM + DSS group (Table S1 and Figure S2C). Among them, 20 commonable differential metabolites (CDMs) existed between the control_2 vs. Veh + DSS and Veh + DSS vs. LCM + DSS (Figure 4B). Thirty exclusive differential metabolites (EDMs) were only observed in the Veh + DSS vs. LCM + DSS. Phenylethylamine, PC(22:5(7Z,10Z,13Z,16Z,19Z)/18:1(11Z)), 2,4-Pentadienal, pyrrolidine, succinic acid, indoleacetic acid, dodecanedioic acid, 5′-Deoxy-5′-methylthioadenosine, (Alpha-D-mannosyl)7-beta-D-mannosyl-diacetylchitobiosyl-L-asparagine, isoform A (protein), and hydroxypyruvic acid were the top 10 significant differential metabolites in Veh + DSS vs. LCM + DSS (Figure 4C and Table S2). Notably, the low dose *CM* powder treatment reversed the up-regulation of eight metabolites and the down-regulation of twelve metabolites of CDMs in DSS-injured mice, when compared to the control_2 group. Twenty-five metabolites were increased and five metabolites were reduced in EDMs, which were not observed in the control_2 vs. Veh + DSS. Additionally, the metabolic pathway analysis of KEGG-enriched pathways showed that significantly differential metabolites of Veh + DSS vs. LCM + DSS involved the pathways of ATP binding cassette (ABC) transporters (19.05%), glycerophospholipid metabolism (14.29%), neuroactive ligand–receptor interaction (9.52%), amino acid metabolism (9.52%), arachidonic acid metabolism (9.52%), galactose metabolism (9.52%), and choline metabolism in cancer (9.52%) (Figure 4D). Based on enrichment analysis and topological analysis, the biosynthesis and degradation of valine, leucine, and isoleucine and the metabolism of tryptophan, phenylalanine, tyrosine, glycine, serine, threonine, starch, sucrose, sphingolipid, glyoxylate, dicarboxylate, and arachidonic were the key pathways with the highest correlation for significantly differential metabolites of Veh + DSS vs. LCM + DSS, particularly amino acid metabolism (Figure 4E). Thus, the *CM* powder treatment affected metabolic pathways significantly.

### 3.5. Correlation Analysis among Gut Microbiota, Metabolites, and Other Parameters in DSS-Injured Mice

**Figure 5:**
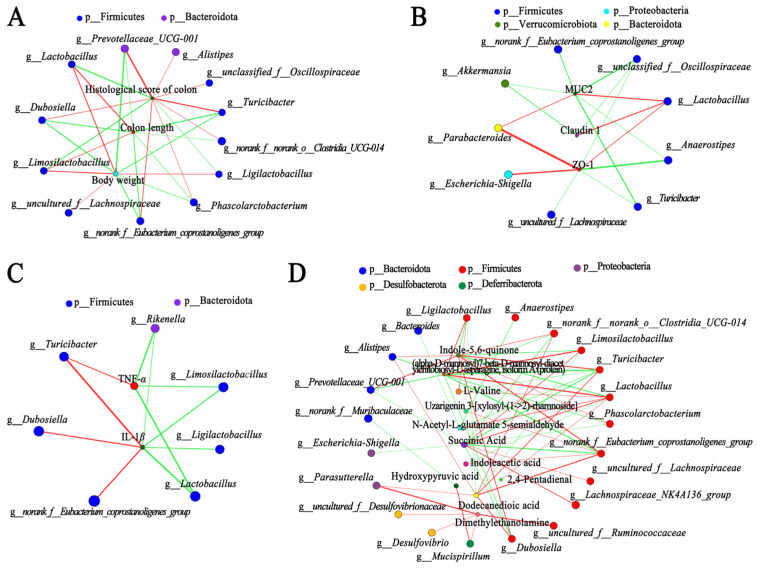
Correlation among gut microbiota, metabolites, and other parameters. (**A**) Network diagram showing relationships between gut microbiota and disease signs. (**B**) Network diagram showing relationships between gut microbiota and indicators of intestinal mucosal barrier. (**C**) Network of correlation analysis between gut microbiota and cytokines. (**D**) Network of correlation analysis between gut microbiota and special metabolites. In Network diagrams, the node size represents species abundance, the line thickness represents the correlation coefficient, the red line represents a positive correlation, and the green line represents a negative correlation. The correlation analysis of Heatmap and Network was carried out by Spearman’s correlation coefficients.

Spearman’s correlation analysis was conducted to explore the potential mechanism. The top 10 significant differential metabolites of CDMs and EDMs were selected as special differential metabolites (Table S2). After removing unqualified factors with variance inflation factors (VIF) > 10 [15], three disease signs, three indicators of the intestinal mucosal barrier, three cytokines, and thirteen special differential metabolites were obtained for analysis (Table S3). Gut microbiota had a significant interactions with disease signs (including body weight, colon length, and the histological score of colons) (Figure 5A) and indicators of the intestinal mucosal barrier (Figure 5B). *Lactobacillus*, *Limosilactobacillus*, and *Ligilactobacillus* were negatively correlated with the HS of colon. *Lactobacillus*, *Limosilactobacillus*, *uncultured_f__Lachnospiraceae*, and *Ligilactobacillus* were positively correlated with body weight and colon length. Moreover, *Turicibacter*, *Dubosiella*, and *norank_f__Eubacterium_coprostanoligenes_group* were positively correlated with TNF-*α* and IL-1*β* (Figure 5C). *Lactobacillus*, *Rikenella*, *Limosilactobacillus*, and *Ligilactobacillus* were negatively correlated with TNF-*α* and IL-1*β* (Figure 5C). As shown in Figure 5D, Firmicutes is the main phylum that was significantly associated with the modulation of metabolites, including *Ligilactobacillus*, *Turicibacter*, *Anaerostipes*, *Lactobacillus*, *norank_f__Eubacterium_coprostanoligenes_group*, *uncultured_f__Ruminococcaceae*, *Dubosiella*, *uncultured_f__Lachnospiraceae*, *Limosilactobacillus*, *norank_f__norank_o__Clostridia_UCG-014*, and *Phascolarctobacterium*. Moreover, *Bacteroides*, *Alistipes*, *Prevotellaceae_UCG-001*, *Escherichia-Shigella*, *uncultured_f__Desulfovibrionaceae*, and *Mucispirillum* also had a clearly positive and/or negative correlation with special differential metabolites. Interestingly, the low dose *CM* powder treatment improved the changes in glycerophospholipids in CDMs (Figure S3A). According to the VIF < 10 of glycerophospholipids (Table S3), Spearman’s correlation analysis of gut microbiota and three glycerophospholipids showed that *uncultured_f__Lachnospiraceae*, *Lactobacillus*, *Limosilactobacillus*, and *Lachnospiraceae_NK4A136_group* were positively correlated with the three glycerophospholipids, respectively (Figure S3B). *Dubosiella*, *Turicibacter*, *Alistipes*, *norank_f__Eubacterium_coprostanoligenes_group*, *Phascolarctobacterium*, *norank_f__norank_o__Clostridia_UCG-014*, and *norank_f__Muribaculaceae* were negatively correlated with the three glycerophospholipids, respectively (Figure S3B). These results implied that the effect of *CM* powder on UC was related to the interaction among gut microbiota, metabolites, and intestinal mucosal barrier.

### 3.6. Peptides and Polysaccharides of CM Changed the Gut Microbiota Structure of UC Patient’s Faeces

**Figure 6:**
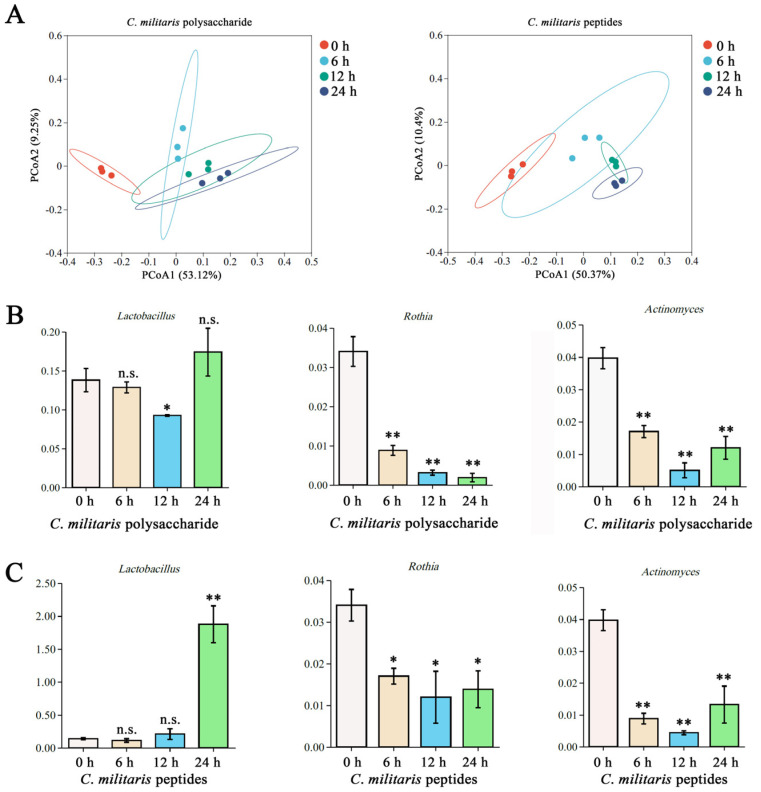
Response of the gut microbiota in UC patient’s faeces to fermentation of *CM* polysaccharides and peptides. (**A**) PCoA of microbial composition. (**B**,**C**) Variation in specific genera by polysaccharide and peptides, including *Lactobacillus*, *Rothia*, and *Actinomyces*. *, *p* < 0.05 versus 0 h group; **, *p* < 0.01 versus 0 h group; n.s., no significant difference (*p* > 0.05).

The major active compounds with a high content of *CM* are polysaccharides (~40%) and proteins/peptides (~30%) [12,14,15,21]. To further investigate the regulatory effect of *CM* on the gut microbiota in humans, the structure of UC patients’ feces gut microbiota was analyzed by fermenting peptides and polysaccharides from *CM*. As showed in Figure 6A, the 0 h group was significantly different from each sample treatment group, and there was no obvious overall region among all experimental groups in PCoA, implying that peptides and polysaccharides of *CM* significantly altered the structure of the gut microbiota during fermentation. Furthermore, peptides and polysaccharides with *CM* intervention enhanced the abundances of *Lactobacillus* after 24 h of fermentation, particularly peptides (*p* < 0.01) (Figure 6B,C). A significant reduction in *Rothia* and *Actinomyces* was observed during fermentation with treatment using peptides and polysaccharides.

## 4. Discussion

The anti-inflammatory effects of edible plants have been reported in various studies [22], while only a few studies focused on the effects of edible mushrooms on UC. This study explored the effect of prophylactic supplementation of *CM* powder on UC in mice. Generally, weight loss, diarrhea, bloody stool, and inflammation levels were indirect markers of UC [23,24]. Inflammation, congestion, and edema of the colon could cause a decrease in colon length which is related to the severity of UC [15,24,25]. Surprisingly, prophylactic supplementation with *CM* powder significantly improved UC-related symptoms, inhibited the increase in TNF-*α* and IL-1*β* in serum, and prevented a decrease in IL-10. Importantly, the animal dose (100 mg/kg BW/day) of *CM* can be extrapolated to a human equivalent theoretical dose of consuming about 0.5 g per day (based on a body weight of 60 kg) [26], which is reasonably achievable in a human diet. Therefore, the inclusion of whole *CM* powder in the diet at an appropriate dose is a potential prebiotic to intervene in UC, encouraging us to further investigate the mechanisms by which whole *CM* powder relieves UC. Undoubtedly, recommended intake levels and toxicologic investigations of *CM* need to be researched in further studies.

Abnormalities in intestinal mucosal barriers are likely an early event in the pathogenesis of UC [8,27]. Dysfunction of intestinal mucosal barriers may increase the permeability, subsequently exacerbating the invasion of intestinal commensal microbes and toxins, resulting in a persistent inappropriate immune response that promotes chronic inflammation [28]. In the present study, *CM* powder improved the adverse symptoms caused by damage to the structure of the intestinal mucosal barrier. Moreover, MUC2 is produced by intestinal goblet cells in the epithelial cell layer and dominated the mucus layer as part of intestinal mucosal barrier [29]. Mucosal inflammation was negatively correlated with the concentration of MUC2 in UC patients [30]. Simultaneously, research has also shown that the intestinal mucosal barrier is mainly regulated by epithelial TJ proteins [20]. Claudin and occludin form homotypic complexes between epithelial cells, and ZO-1 connects occludin and claudin to the actin cytoskeleton, in order to maintain the integrity of the structure and the normal functioning of the intestinal mucosal barrier [31]. Taken together, the loss of MUC2 and TJ proteins may be key to the destruction of the intestinal mucosal barrier destruction in this study. Thus, prophylactic supplementation with *CM* powder effectively mitigates the impairment of the intestinal mucosal barrier in DSS-induced UC, by up-regulating MUC2 and TJ proteins.

Some studies have reported that gut microbiota dysbiosis is associated with the pathogenesis and development of UC [6,32]. The gut microbiota mediated the intestinal immunity response, inflammatory response, and intestinal mucosal barrier function to intervene in UC [33]. Diet is one of the inevitable factors affecting gut microbiota, which is closely related to UC. There were no statistically significant differences between the model group and the sample treatment groups in gut microbiota composition at T1 (Figure 3A), while the *CM* powder treatment enhanced the abundance of beneficial bacteria (*Lactobacillus*, *Odoribacter*, and *Mucispirillum*) in DSS-injured mice at T2, implying that prophylactic treatment with *CM* powder subsequently affects the gut microbiota structure during DSS exposure. More importantly, *Lactobacillus* is one of the probiotics that maintains intestinal health, as it can regulate intestinal homeostasis, host immunity, and activation of the aryl hydrocarbon receptor (AhR) pathway against intestinal mucosal barrier damage in UC [34,35]. *Mucispirillum* and *Odoribacter* are protective species to improve UC symptoms [36,37]. *Mucispirillum* can trigger T-cell-dependent immunoglobulin A and immunoglobulin G, and their subsequent immune responses [36,38]. *Odoribacter* have an ability to protect cells from UC [37,39]. Moreover, short-chain fatty acids (SCFAs) are a well-known bacterial metabolite with anti-inflammatory effects. *Lactobacillus* and *Odoribacter* can produce SCFAs to exert their probiotic effects on the intestinal mucosal barrier [15,37,40]. Furthermore, the integrity of the intestinal mucosal barrier depends on its interactions with gut microbiota. The impairment of the intestinal mucosal barrier is closely associated with the uncontrollable immune reaction and/or the unrestricted dysbiosis of gut microbiota in the intestine [41]. The gut microbiota at the outer mucus layer not only modulates mucus layer dynamics, but also mediates mucin production and secretion to maintain mucus barrier integrity [42]. *Lactobacillus* can strengthen the functions of the intestinal mucosal barrier, through enhancing goblet cells and Paneth cells, promoting the formation and expression of TJs, and increasing immune barrier functions [43]. *Mucispirillum* is involved as a mucus associated niche in the distal colon, which may be important to forming a functional protective intestinal mucus layer [44]. Zhao et al. found that an increase in *Odoribacter* is positively correlated with the intestinal mucosal barrier and inflammatory responses, contributing to attenuating DSS-induced colitis [45]. Summarily, the regulating properties of gut microbiota and their potential effects on intestinal mucosal barrier function are the key to *CM* powder resisting UC.

Gut microbiota-derived metabolites have important effects on metabolic and nutritional homeostasis and immune system maturation and stimulation [34]. Microbial metabolites are the connecting links between gut microbiota and the host’s health in UC, as they modulate immune and metabolic responses to interferences with gut homeostasis and disease progression [32]. The correlation analysis displays significant interactions between gut microbiota and metabolites in this study. Amino acid metabolism is an important enrichment pathway of significantly differential metabolites. Previous research has proved that the feces of patients with UC had different degrees of changes in various amino acids, such as a reduction in valine and glutamate expression and an enhancement of tryptophan and isoleucine content [46,47]. Generally, the difference in microbial metabolites in DSS-injured mice are mainly concentrated in amino acid metabolism, which may contribute to the differential expressions of mucin and mucus [47]. Zhu et al. demonstrated that a Tanshinone IIA treatment mainly improved DSS-induced UC by altering the metabolisms of amino acid, arachidonic acid, glyoxylate, and dicarboxylate [48]. Our metabolomics analysis also is consistent with these results. Thus, the microbiota–metabolites axis is one of the critical pathways for *CM* powder against UC.

It should be noted that the abundance of *Lactobacillus* was obviously enhanced by *CM* powder. *Lactobacillus* was not only negatively correlated with TNF-*α*, IL-1*β*, and the HS of the colon, but was also positively correlated with MUC2, claudin 1, and ZO-1. Furthermore, the enrichment metabolic pathway involved tryptophan metabolism in this study. Accumulating data suggest that *Lactobacillus* metabolizes dietary tryptophan to activate the aromatic hydrocarbon receptor pathway; this may be an important way to regulate intestinal homeostasis and host immunity, including gut barrier protection and immune modulation [34]. Interestingly, oral administration of *CM* powder could directly improve the DSS-induced change in glycerophospholipids, which has a clear correlation with gut microbiota. Yuan et al. reported that Huang-Lian Jie-Du decoction mainly modulates the arachidonic acid metabolic pathway and the glycerophospholipid metabolic pathway to alleviate UC, which are involved in inhibiting cyclooxygenase-2 protein expression and the activity of phospholipase A2 and 5-lipoxygenase [49]. These findings suggested that the effects of *CM* powder on the microbiota–metabolites axis in UC are worth further study in the future, particularly focusing on the pathway of *Lactobacillus* and the metabolic pathways of amino acid and glycerophospholipid in repairing the intestinal mucosal barrier.

In fact, the biological activity of edible plants is closely related to their active compounds. The total content of protein and polysaccharides were >70% in *CM* [12,21]. In the present study, an in vitro fermentation model revealed that the gut microbiota structure was changed significantly by the peptides and polysaccharides of *CM*, which may be a major factor for the effect of the regulatory properties of *CM* on the gut microbiota. Notably, *Rothia* and *Actinomyces* could be considered biomarkers in patients with active UC [50]. A lower level of *Lactobacillus* and higher levels of *Rothia* and *Actinomyces* in patients with active UC, compared to in healthy controls, were reported in a clinical study [50]. In contrast, our results found that peptides and polysaccharides from *CM* enhanced *Lactobacillus* (beneficial bacteria) and reduced *Rothia* and *Actinomyces* (harmful bacteria) in UC patients’ feces. These results suggest that peptides and polysaccharides from *CM* had the ability to regulate gut microbiota in the fecal samples of UC patients. The effects of the regulatory properties of *CM* on gut microbiota in UC may be the result of the potential synergistic effects of peptides and polysaccharides. Our data provide further evidence for the effects of the regulatory properties of *CM* on gut microbiota and the efficacy of *CM* as a dietary supplement for UC prevention in humans.

## 5. Conclusions

The present research demonstrated that prophylactic treatment of *CM* could effectively mitigate gut microbiota dysbiosis and intestinal mucosal barrier damage in DSS-induced UC mice. Further analysis showed that *CM* regulates gut microbiota and subsequently affects metabolic pathways, which may be involved in the intestinal mucosal barrier repair in order to alleviate UC, involving the abundance of *Lactobacillus*, *Odoribacter*, and *Mucispirillum*, and the metabolic pathways of amino acid metabolism, glyoxylate and dicarboxylate metabolism, and arachidonic acid metabolism. Furthermore, peptides and polysaccharides from *CM* can alter gut microbiota structure in feces samples from UC patients. In summary, our results reveal that *CM* could act as a potential and economical prebiotics to improve UC, via modulating the microbiota–metabolites axis and repairing the intestinal mucosal barrier, which also provides a holistic perspective for dietary intake and gut microbiota homeostasis in the disease progression of UC.

## Figures and Tables

**Table 1:** Disease activity index (DAI) scoring criteria.

Body Weight Loss	Stool Consistency for Diarrhea	Fecal Occult Blood	Score
None	Normal	Normal	0
1–5%	Soft but still formed	Melena and negative blood	1
6–10%	Soft	Positive blood	2
11–15%	Soft and wet	Blood stains can be seen on the stool	3
>15%	Watery diarrhea	Rectal bleeding	4

The DAI is expressed as the average of these scores.

**Table 2:** Histological score (HS) criteria.

Inflammation	Mucosal Damage	Crypt Loss	Pathological Change Range	Score
None	None	None	None	0
Mild	Mucus layer	1–33%	1–25%	1
Moderate	Submucosa	34–66%	26–50%	2
Severe	Muscular and serosa	67–100% + intact epithelium	51–75%	3
-	-	100% with epithelium lose	76–100%	4

The HS is expressed as the average of these scores.

## Data Availability

The data presented in this study are available on request from the corresponding author. The data are not publicly available due to respect of patient privacy.

## Supplementary Materials

The following supporting information can be downloaded at: https://www.mdpi.com/article/10.3390/nu15204378/s1; Figure S1: differences in the composition of gut microbial and diversity analysis at T1 and T2; Figure S2: metabonomic analysis of faeces; Figure S3: correlation analysis of glycerophospholipids; Table S1: the statistical information of differential metabolisms among different groups under positive and negative electrospray ionization modes (VIP > 1 and *p* < 0.05); Table S2: detailed information for significantly different metabolites; Table S3: variance inflation factor (VIF) of different environmental factors for correlation analysis.
