# Supplementary material for: Effect of Cordyceps militaris Powder Prophylactic Supplementation on Intestinal Mucosal Barrier Impairment and Microbiota-Metabolites Axis in DSS-Injured Mice
Source: Nutrients. 2023 Oct 16;15(20):4378. doi: 10.3390/nu15204378 (PMC10610503; doi:10.3390/nu15204378)
Supplement: Supplementary file 1 [file nutrients-15-04378-s001.zip › nutrients-2621798-supplementary.pdf]

Table S1 The statistical information of differential metabolism among different groups under positive and negative electrospray ionization mode (VIP > 1 and $p < 0.05$)

**Group**	**differential metabolism**	**Up-regulated**	**down-regulated**
Control_2 vs Veh+DSS	167	69	98
Veh+DSS vs LCD+DSS	50	37	13

Table S2 The detail information for significant different metabolites

Name	Mw (g/mol)	Rt	m/z	Super.Class	Class
Succinic acid	118.09	225.80	117.02	/	/
Hydroxypyruvic acid	104.06	228.34	103.00	Organic acids and derivatives	Hydroxy acids and derivatives
Indoleacetic acid	175.18	58.10	174.06	Organoheterocyclic compounds	Indoles and derivatives
(Alpha-D-mannosyl)7-beta-D-mannosyl-diacetylchitobiosyl-L-asparagine, isoform A (protein)	89.09	155.36	90.05	Organic acids and derivatives	Carboxylic acids and derivatives
2,4-Pentadienal	82.10	227.22	83.05	Organic oxygen compounds	Organooxygen compounds
Dodecanedioic acid	230.30	173.84	229.15	Lipids and lipid-like molecules	Fatty Acyls
PC(22:5(7Z,10Z,13Z,16Z,19Z)/18:1(11Z))	834.20	21.10	834.60	Lipids and lipid-like molecules	Glycerophospholipids
Sphinganine	301.50	42.81	302.30	Organic nitrogen compounds	Organonitrogen compounds
PC(22:4(7Z,10Z,13Z,16Z)/16:0)	810.10	21.20	810.60	Lipids and lipid-like molecules	Glycerophospholipids
PC(22:5(4Z,7Z,10Z,13Z,16Z)/16:0)	808.10	39.79	808.58	Lipids and lipid-like molecules	Glycerophospholipids
5-Aminovaleric acid	117.15	227.34	100.08	/	/
Alpha-Amino-4-carboxy-3-furanpropanoic acid	199.16	27.76	200.05	/	/
2-Ethylacrylic acid	100.12	227.38	101.06	Lipids and lipid-like molecules	Fatty Acyls
L-Valine	117.15	227.38	118.09	Organic acids and derivatives	Carboxylic acids and derivatives
Palmitoylethanolamide	299.50	33.98	300.29	Organic acids and derivatives	Carboximidic acids and derivatives
PC(22:5(7Z,10Z,13Z,16Z,19Z)/16:0)	808.10	20.52	808.58	Lipids and lipid-like molecules	Glycerophospholipids

Indole-5,6-quinone	147.13	199.18	148.04	Organoheterocyclic compounds	Indoles and derivatives
Dimethylethanolamine	89.14	183.63	90.09	Organic nitrogen compounds	Organonitrogen compounds
Salsolinol	179.22	106.88	180.10	Organoheterocyclic compounds	Tetrahydroisoquinolines
Uzarigenin 3-[xylosyl-(1->2)-rhamnoside]	652.80	156.48	653.35		
PC(18:2(9Z,12Z)/P-18:0)	652.80	33.80	770.61	Lipids and lipid-like molecules	Glycerophospholipids
PC(22:6(4Z,7Z,10Z,13Z,16Z,19Z)/22:6(4Z,7Z,10Z,13Z,16Z,19Z))	878.20	20.53	878.57	Lipids and lipid-like molecules	Glycerophospholipids
N-Acetyl-L-glutamate 5-semialdehyde	173.17	219.11	174.08	Organic acids and derivatives	Carboxylic acids and derivatives
Prostaglandin D2	352.50	166.55	351.22	Lipids and lipid-like molecules	Fatty Acyls

Mw: Molecular weight

m/z: Mass to charge ratio

Rt: Retention times

Super. Class: Secondary classification in the HMDB database

Class: Tertiary classification in the HMDB database

Table S3 Variance inflation factor (VIF) of different environmental factors for correlation analysis

Environmental factors	VIF	Environmental factors	VIF	Environmental factors	VIF
Body weight	3.46	N-Acetyl-L-glutamate 5-semialdehyde	1.34	Uzarigenin 3-[xylosyl-(1->2)-rhamnoside]	4.92
DAI	55.46	Prostaglandin D2	3.17	2-Ethylacrylic acid	49.68
Colon length	5.21	Indoleacetic acid	2.22	5-Aminovaleric acid	20.83
Histological score of colon	5.50	Dodecanedioic acid	2.60	Alpha-Amino-4-carboxy-3-furanpropanoic acid	18.57
MUC2	2.67	Dimethylethanolamine	2.13	Sphinganine	63.06
Claudin 1	5.72	Hydroxypyruvic acid	2.16	Salsolinol	19.50
Occludin	41.49	L-Valine	3.50	PC(22:4(7Z,10Z,13Z,16Z)/16:0)	40.68
ZO-1	5.93	(Alpha-D-mannosyl)7-beta-D-mannosyl-diacetylchitobiosyl-L-asparagine, isoform A (protein)	3.23	PC(22:5(4Z,7Z,10Z,13Z,16Z)/16:0)	85.92
TNF-α	3.30	2,4-Pentadienal	2.45	PC(22:5(7Z,10Z,13Z,16Z,19Z)/16:0)	44.59
IL-1β	3.07	Indole-5,6-quinone	2.67	PC(22:5(7Z,10Z,13Z,16Z,19Z)/18:1(11Z))	6.78
IL-10	1.60	Palmitoylethanolamide	4.41	PC(18:2(9Z,12Z)/P-18:0)	1.51
		Succinic acid	2.55	PC(22:6(4Z,7Z,10Z,13Z,16Z,19Z)/22:6(4Z,7Z,10Z,13Z,16Z,19Z))	7.51

Figure S1 Differences in the composition of gut microbial and diversity analysis at T1 and T2. (A, B) The Rank-abundance curves and Shannon index for rarefaction curves at OTU level of groups. (C, D) The coverage index of OTU level at T1 and T2. (E) Venn diagram of OTU levels at T2. (F) The community bar plots at the genus level at T2.

Figure S2 Metabonomics analysis of faeces. (A) The permutation histogram test of OPLS-DA model. (B) The principal component analysis. (C) Pie chart of metabolite classification and proportion. (D) The volcano plot of differential metabolism in two groups' comparisons.

Figure S3 Correlation analysis glycerophospholipids. (A) Changes in differential glycerophospholipids. (B) Network diagram showing relationships between gut microbiota and several glycerophospholipids. In Network, node size represents species abundance, line thickness represents the correlation coefficient, the red line represents a positive correlation, and the green line represents a negative correlation. The correlation analysis of Network was carried out by Spearman's correlation coefficients.

Figure S1

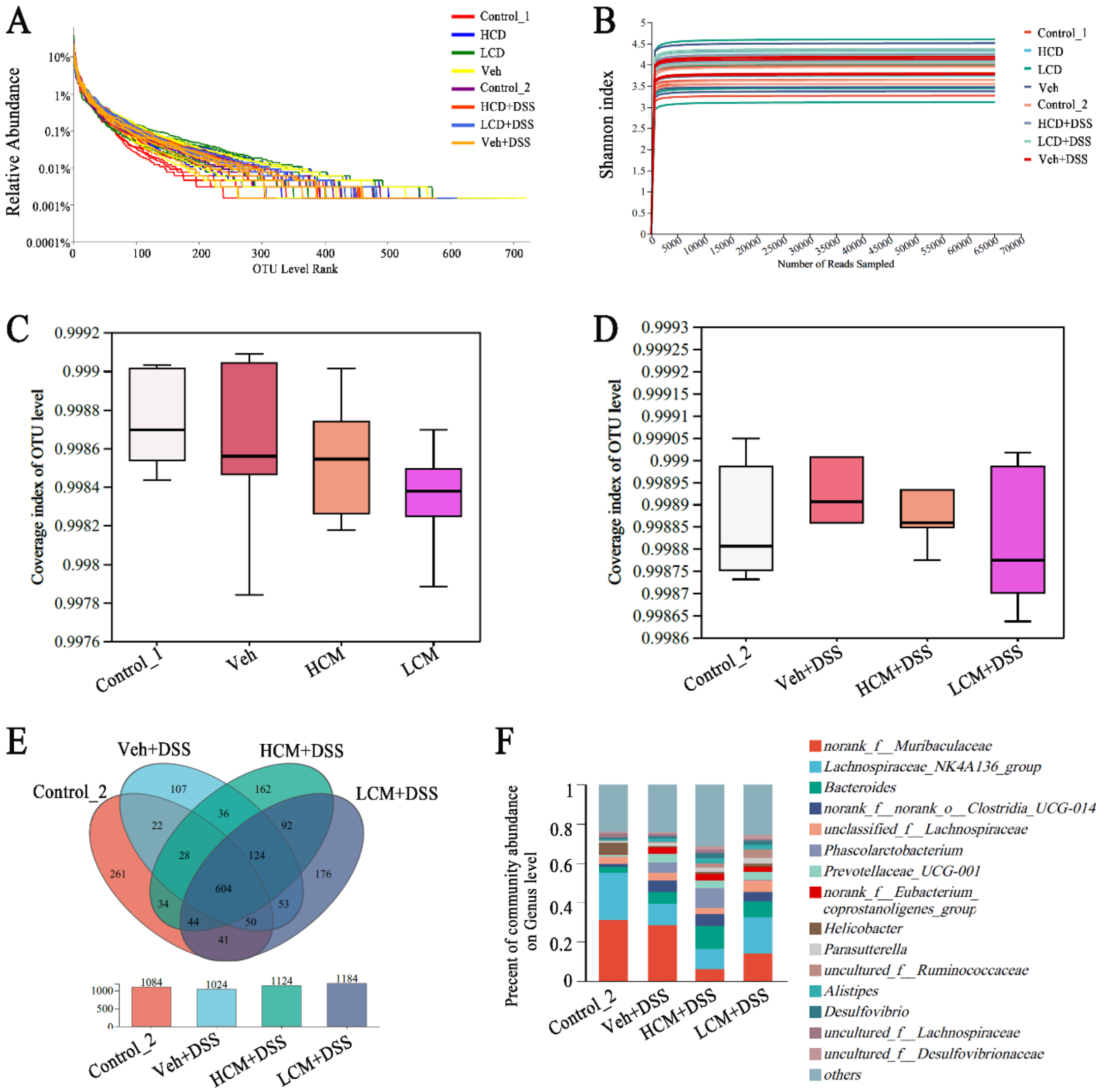
A
Relative Abundance
OTU Level Rank
Control_1
HCD
LCD
Veh
Control_2
HCD+DSS
LCD+DSS
Veh+DSS
B
Shannon index
Number of Reads Sampled
C
Coverage index of OTU level
Control_1
Veh
HCM
LCM
D
Coverage index of OTU level
Control_2
Veh+DSS
HCM+DSS
LCM+DSS
E
Control_2
Veh+DSS
HCM+DSS
LCM+DSS
F
Precent of community abundance on Genus level
norank_f__Muribaculaceae
Lachnospiraceae_NK4A136_group
Bacteroides
norank_f__norank_o__Clostridia_UCG-014
unclassified_f__Lachnospiraceae
Phascolarctobacterium
Prevotellaceae_UCG-001
norank_f__Eubacterium_coprostanoligenes_group
Helicobacter
Parasutterella
uncultured_f__Ruminococcaceae
Alistipes
Desulfovibrio
uncultured_f__Lachnospiraceae
uncultured_f__Desulfovibrionaceae
others

Figure S2

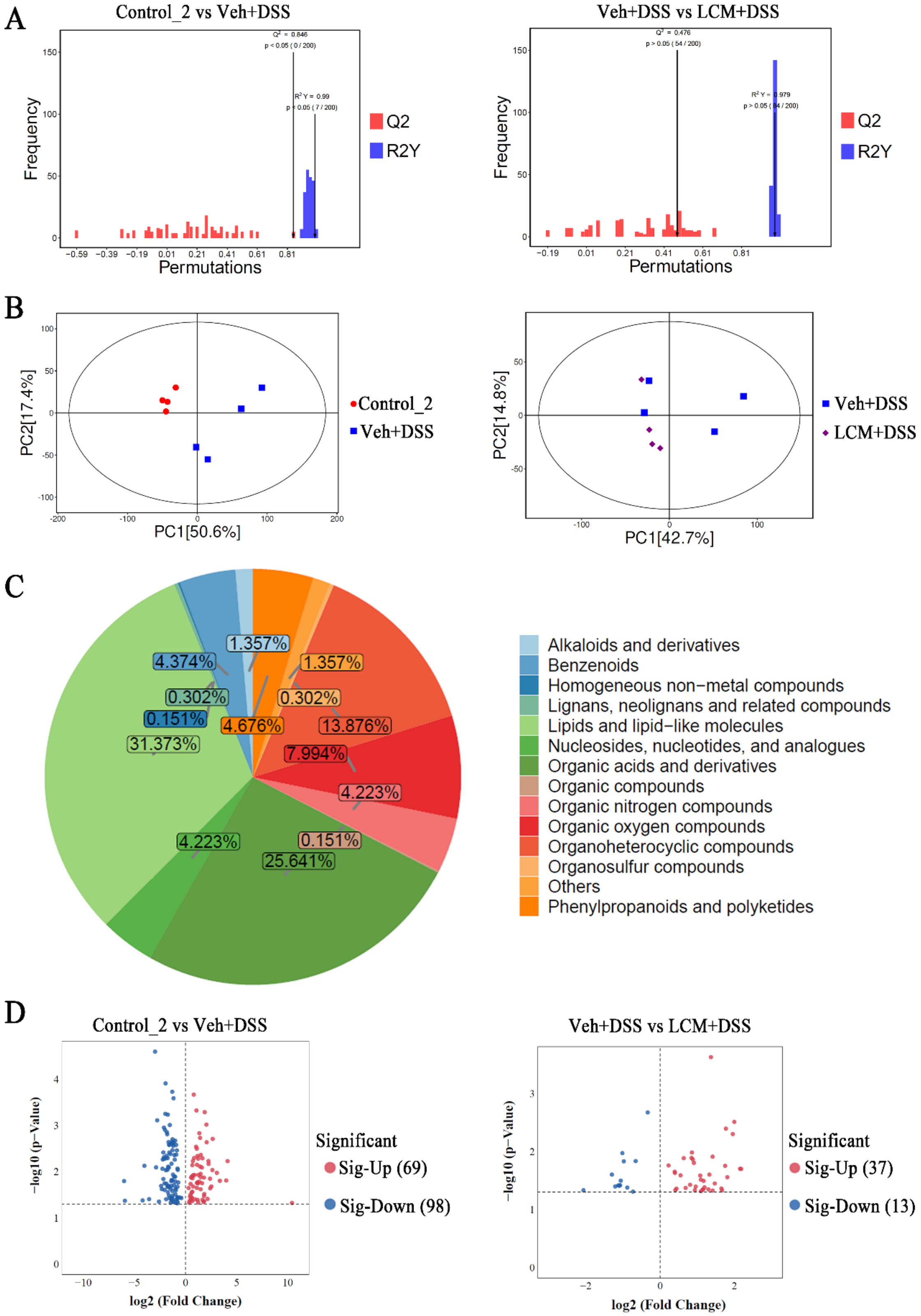
A
Control_2 vs Veh+DSS
Frequency
Permutations
Q2
R2Y
Veh+DSS vs LCM+DSS
B
PC2[17.4%]
PC1[50.6%]
Control_2
Veh+DSS
PC2[14.8%]
PC1[42.7%]
LCM+DSS
C
Alkaloids and derivatives
Benzenoids
Homogeneous non−metal compounds
Lignans, neolignans and related compounds
Lipids and lipid−like molecules
Nucleosides, nucleotides, and analogues
Organic acids and derivatives
Organic compounds
Organic nitrogen compounds
Organic oxygen compounds
Organoheterocyclic compounds
Organosulfur compounds
Others
Phenylpropanoids and polyketides
1.357%
4.374%
0.302%
0.151%
31.373%
4.676%
13.876%
7.994%
4.223%
4.223%
0.151%
25.641%
D
Control_2 vs Veh+DSS
−log10 (p−Value)
log2 (Fold Change)
Significant
Sig-Up (69)
Sig-Down (98)
Veh+DSS vs LCM+DSS
Sig-Up (37)
Sig-Down (13)

Figure S3

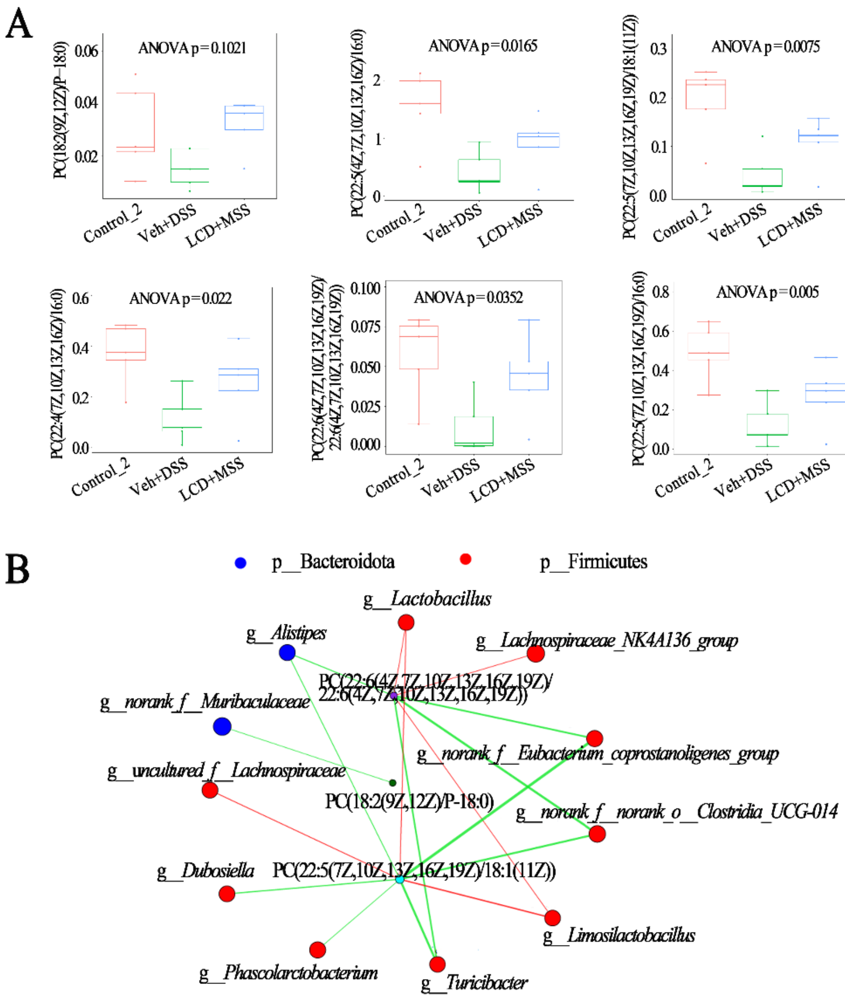
A
ANOVA p=0.1021
PC(18:2(9Z,12Z)/P-18:0)
ANOVA p=0.0165
PC(22:5(4Z,7Z,10Z,13Z,16Z)/16:0)
ANOVA p=0.0075
PC(22:5(7Z,10Z,13Z,16Z,19Z)/18:1(11Z))
ANOVA p=0.022
PC(22:4(7Z,10Z,13Z,16Z)/16:0)
ANOVA p=0.0352
PC(22:6(4Z,7Z,10Z,13Z,16Z,19Z)/22:6(4Z,7Z,10Z,13Z,16Z,19Z))
ANOVA p=0.005
PC(22:5(7Z,10Z,13Z,16Z,19Z)/16:0)
Control_2
Veh+DSS
LCD+MSS
B
p__Bacteroidota
p__Firmicutes
g__Lactobacillus
g__Alistipes
g__Lachnospiraceae_NK4A136_group
PC(22:6(4Z,7Z,10Z,13Z,16Z,19Z)/22:6(4Z,7Z,10Z,13Z,16Z,19Z))
g__norank_f__Muribaculaceae
g__norank_f__Eubacterium_coprostanoligenes_group
g__uncultured_f__Lachnospiraceae
PC(18:2(9Z,12Z)/P-18:0)
g__norank_f__norank_o__Clostridia_UCG-014
g__Dubosiella
PC(22:5(7Z,10Z,13Z,16Z,19Z)/18:1(11Z))
g__Limosilactobacillus
g__Phascolarctobacterium
g__Turicibacter
